# Pediatric Orbital Langerhans Cell Histiocytosis With Dural Adherence Requiring Orbital Roof Reconstruction: A Case Report

**DOI:** 10.7759/cureus.117742

**Published:** 2026-10-10

**Authors:** Christopher Branstetter, Robert M Branstetter, Brandon Byrd, Oritsejolomi Roberts, Mohamad Masoumy

**Affiliations:** 1 School of Medicine, Louisiana State University Health Sciences Center New Orleans, New Orleans, USA; 2 Department of Ophthalmology, Louisiana State University Health Sciences Center New Orleans, New Orleans, USA; 3 Department of Surgery, Division of Plastic and Reconstructive Surgery, Louisiana State University, New Orleans, USA; 4 Department of Neurosurgery, Louisiana State University Health Sciences Center New Orleans, New Orleans, USA; 5 Department of Surgery, Division of Plastic and Reconstructive Surgery, Louisiana State University Health Sciences Center New Orleans, New Orleans, USA

**Keywords:** dural involvement, langerhans cell histiocytosis(lch), orbital roof reconstruction, pediatric orbital tumor, superolateral orbital mass

## Abstract

We present a rare case describing the clinical course of a seven-year-old girl presenting with left periorbital swelling, initially managed as preseptal cellulitis. The periorbital swelling was unresponsive to multiple courses of antibiotics. Magnetic resonance imaging (MRI) of the head and orbits revealed a 2.3 × 2.4 × 2.1 cm mass involving the superolateral aspect of the left orbit. The lesion was predominantly hypointense on T1-weighted images and demonstrated mixed signal intensity on T2-weighted images. Post-contrast imaging demonstrated peripheral enhancement with internal septations.

Concerns for mass effect due to the tumor size and location prompted surgical resection, with histopathologic examination confirming the diagnosis of Langerhans cell histiocytosis (LCH). Given the central nervous system (CNS)-risk orbital location, the patient completed a 52-week course of vinblastine and prednisone, consistent with a standard 12-month regimen. At the 12-month clinical follow-up after completion of chemotherapy, she remained clinically stable without evidence of recurrence. This case highlights the importance of considering rare diagnoses in pediatric patients with atypical or nonresponsive symptoms, particularly when clinical or laboratory findings do not support an infectious etiology.

## Introduction

Langerhans cell histiocytosis (LCH) is a rare clonal proliferative disorder of dendritic cells, most commonly affecting the skeleton in young children [1]. With typical diagnosis at age 3, reported rates of LCH vary between 2.6 and 8.9 per million children each year under the age of 15 [2]. Although predominantly observed in the pediatric population, orbital LCH has also been reported in adults [3]. LCH most commonly manifests as a solitary lesion frequently involving the orbital roof; however, orbital LCH accounts for fewer than 1% of orbital masses [1,2,4,5]. Clinical presentation may mimic infections or other inflammatory conditions and most commonly includes periorbital swelling, eyelid edema, or proptosis [6]. LCH can range from isolated unifocal bone disease to multisystem involvement, with treatment intensity determined by the extent and location of disease and the presence of risk-organ or CNS-risk involvement [7]. In orbital LCH, lesions involving craniofacial bones with intracranial soft-tissue extension are considered CNS-risk because of their association with subsequent CNS complications [7].

Reports of intracranial extension in the literature are infrequent. Individual case reports have described orbital lesions with intracranial extension [8,9]. Furthermore, direct dural involvement is rarely documented. Zhu et al. reported a dural-origin LCH in a 13-year-old, and Mishra et al. described minimal dural attachment in a skull lesion of a 10-year-old patient [10,11]. A cranial series of 23 children reported that dural invasion was present in two of 16 resections [12]. The largest series of intracranial extension includes those by Singh et al., who described five of eight pediatric orbital cases with anterior cranial fossa involvement, and Lakatos et al., who identified four cases among 31 children and noted frequent dural tail signs on MRI without operative confirmation [4,6].

In the cases of dural involvement, larger (>3 cm) defects were repaired with titanium mesh, and smaller ones were left to remodel spontaneously [12]. In many pediatric orbital lesions, management is conservative with biopsy, curettage, or intralesional steroids while leaving roof defects undisturbed to remodel on their own [5,6,13]. Additionally, for isolated lesions of the calvarium, nonoperative management is well-established [14]. While rigid reconstruction is occasionally reported in pediatric cranial LCH, repair of the orbital roof is not routinely described [12].

Treatment strategies for LCH depend on the site of disease, extent of organ involvement, and the number of systems affected [7]. A 2024 systematic review of 228 orbital LCH cases described heterogeneous treatment approaches, including surgery, corticosteroids, chemotherapy, radiotherapy, and observation, reflecting variation in disease extent and clinical presentation [15]. Pediatric LCH guidelines classify orbital bone lesions with intracranial soft-tissue extension as CNS-risk lesions and indicate systemic therapy for orbital lesions involving adjacent soft tissues [7]. Chemotherapy may also be considered for orbital lesions with dural involvement [3].

In this report, we present the case of a seven-year-old girl with orbital LCH characterized by acute onset, intracranial extension, intraoperatively observed dural adherence, and the need for orbital roof reconstruction. The orbital bone involvement with intracranial extension supported CNS-risk classification, rather than dural adherence alone. This case illustrates individualized surgical and systemic management of orbital LCH with intracranial extension.

## Case presentation

A seven-year-old female with no significant medical history presented to her pediatrician after three days of left periorbital swelling and itching. She was afebrile and otherwise well with normal extraocular movements and an otherwise unremarkable physical examination. She was treated with antihistamines, oral antibiotics, and topical eye drops for presumed cellulitis, with transient improvement followed by recurrence three days later. Oral trimethoprim-sulfamethoxazole (TMP-SMX) and ophthalmic moxifloxacin were then initiated with marked improvement; however, 14 days after symptom onset, the swelling returned. She was subsequently referred to a local ophthalmology clinic, where MRI of the head and orbits with intravenous gadolinium contrast was performed 24 days after symptom onset.

Her orbital MRI demonstrated a 2.3 × 2.4 × 2.1 cm lesion in the superolateral aspect of the left orbit that was predominantly hypointense on T1-weighted images and exhibited mixed signal intensity on T2-weighted images (Figure 1). Post-contrast imaging demonstrated peripheral enhancement with internal septations. The lesion exerted mass effect on the superolateral aspect of the left globe and the superior margin of the lacrimal gland. Additional enhancement was noted in the meninges along the inferior margin of the left frontal lobe and within the subcutaneous tissues overlying the left frontal bone. Imaging also suggested extension of the lesion through the roof of the left orbit. She was transferred and admitted to the nearest children’s hospital on the same day following imaging results. IV ceftriaxone was initiated but discontinued after normal WBC, ESR, and CRP values, and the absence of fever suggested a noninfectious process. Initial exam demonstrated intact cranial nerves with no focal neurological deficits.

**Figure 1:**
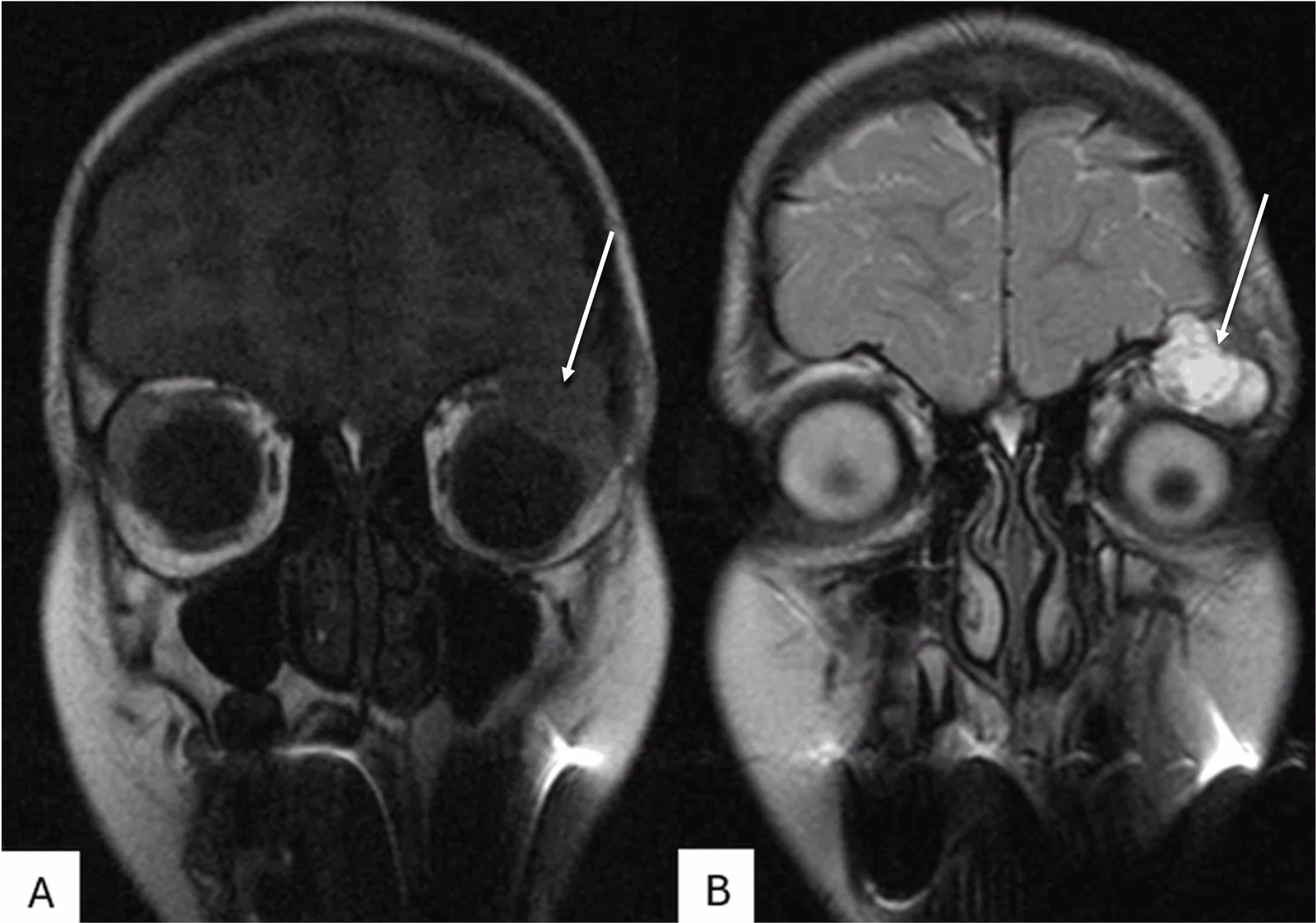
Preoperative magnetic resonance imaging of the head and orbits. (A) Coronal T1-weighted image demonstrating a predominantly hypointense lesion within the superolateral aspect of the left orbit. (B) Coronal T2-weighted image demonstrating heterogeneous signal intensity within the lesion.

Visual acuity was 20/20 in both eyes. Intraocular pressure was 14 mmHg in the right eye and 21 mmHg in the left eye. Pupils were equal, round, and reactive to light, without a relative afferent pupillary defect. Visual fields were full, extraocular movements were full, and ocular alignment was orthophoric. Color vision was intact, with 15/15 Ishihara plates identified in each eye. External examination was normal on the right. On the left, there was hypoglobus and mild enophthalmos, with Hertel exophthalmometry measurements of 17 mm on the right and 15 mm on the left at a base of 88 mm. A 2 × 2 cm left upper eyelid swelling was firm, fixed to the underlying tissue, and nontender, without erythema or warmth. The remainder of the slit-lamp examination was normal bilaterally. Dilated fundus examination was normal in both eyes, with cup-to-disc ratios of 0.25 bilaterally.

Hematology/oncology was consulted to evaluate for multisystem involvement and CNS-risk complications. Additional investigations included a bone scan and abdominal ultrasound, both reported as normal. Baseline laboratory evaluation included a complete blood count with differential, comprehensive metabolic panel, thyroid function testing, and serum and urine osmolality, all of which were within normal limits. Serum sodium, obtained as part of the comprehensive metabolic panel, was normal, and there was no laboratory or clinical evidence of diabetes insipidus at presentation.

Following multidisciplinary review by neurosurgery, plastic surgery, ophthalmology, and hematology/oncology, surgical resection was recommended at a tumor board meeting.

On day four of hospital admission, 28 days after symptom onset, the patient underwent surgical tumor resection with plastic surgery and neurosurgery through a zigzag coronal approach. Following elevation of an anteriorly based pericranial flap, the lesion was exposed within the superolateral orbit and found to contain inflamed tissue and murky fluid. The mass was carefully dissected from the surrounding eyelid soft tissues, and areas of abnormal orbital roof bone were debrided and curetted. The lesion was noted to extend to the dura and was separated from the underlying dura using a combination of sharp and blunt dissection. Following complete excision, a greater than 1 cm full-thickness defect of the orbital roof remained. Because the diagnosis was not yet definitive intraoperatively, and given concern for maintaining the anatomic separation between the orbit and intracranial compartment and preventing herniation of intracranial contents into the orbit, the defect was reconstructed using a preformed three-dimensional titanium cranial mesh plate (Figure 2). The vascularized pericranial flap was then repositioned over the reconstruction to provide coverage of the hardware. The scalp was reflected, and forced duction showed no restriction. The wound was then closed in layers over a surgical drain, and postoperative recovery was stable.

**Figure 2:**
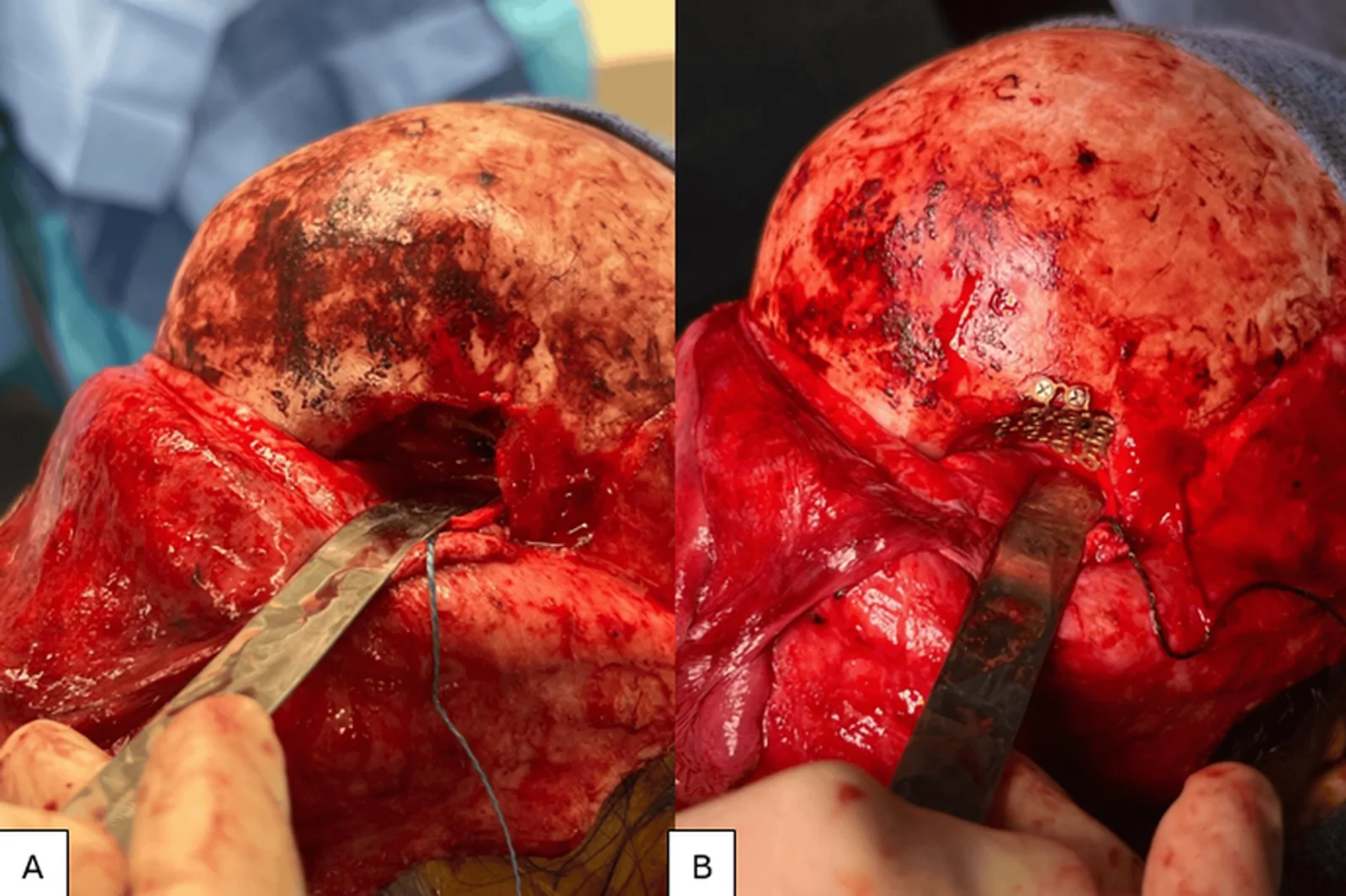
Intraoperative sequence of orbital LCH resection and orbital roof reconstruction. (A) Orbital roof defect following anterior reflection of the scalp and tumor resection. (B) Reconstruction of the orbital roof defect with a cranial mesh plate.

Pathology demonstrated a lesion composed of histiocytes, multinucleated giant cells, eosinophils, and lymphocytes. The histiocytes showed characteristic grooved and folded nuclei with fine chromatin, inconspicuous nucleoli, and minimal cytologic atypia. Associated bony invasion, fibrosis, and destruction of the normal marrow architecture were present. Mitotic activity was increased with no atypical mitoses noted, and the Ki-67 index was 40%. Immunohistochemical staining demonstrated positivity for CD1a and S100, confirming the diagnosis of LCH. The lesional cells were negative for CD30, alpha-smooth muscle actin (SMA), ALK1, desmin, PAX-5, CD99, synaptophysin, and CD3, with only focal positivity for CD15. BRAF V600E testing was negative. Langerin (CD207) immunostaining was not performed.

Given the orbital bone involvement with intracranial extension, systemic therapy with vinblastine and prednisone was initiated by hematology/oncology. The initial treatment phase consisted of six vinblastine doses administered at approximately weekly intervals, together with prednisone at 15 mg each morning and 20 mg each evening for 28 days, followed by a taper. MRI after six weeks demonstrated no evidence of disease. The patient then proceeded to continuation therapy administered in 21-day cycles, consisting of vinblastine 6 mg/m² on day 1 and prednisone 40 mg/m²/day on days 1-5 of each cycle. She completed the full 52-week treatment course.

Her most recent surveillance MRI (Figure 3), obtained approximately four months after completion of chemotherapy and 16 months after surgery, demonstrated no evidence of recurrence. At 12-month clinical follow-up after completion of chemotherapy, she remained clinically stable without evidence of recurrence. She continues to be monitored by the hematology/oncology team every three months with annual imaging for evaluation of recurrence.

**Figure 3:**
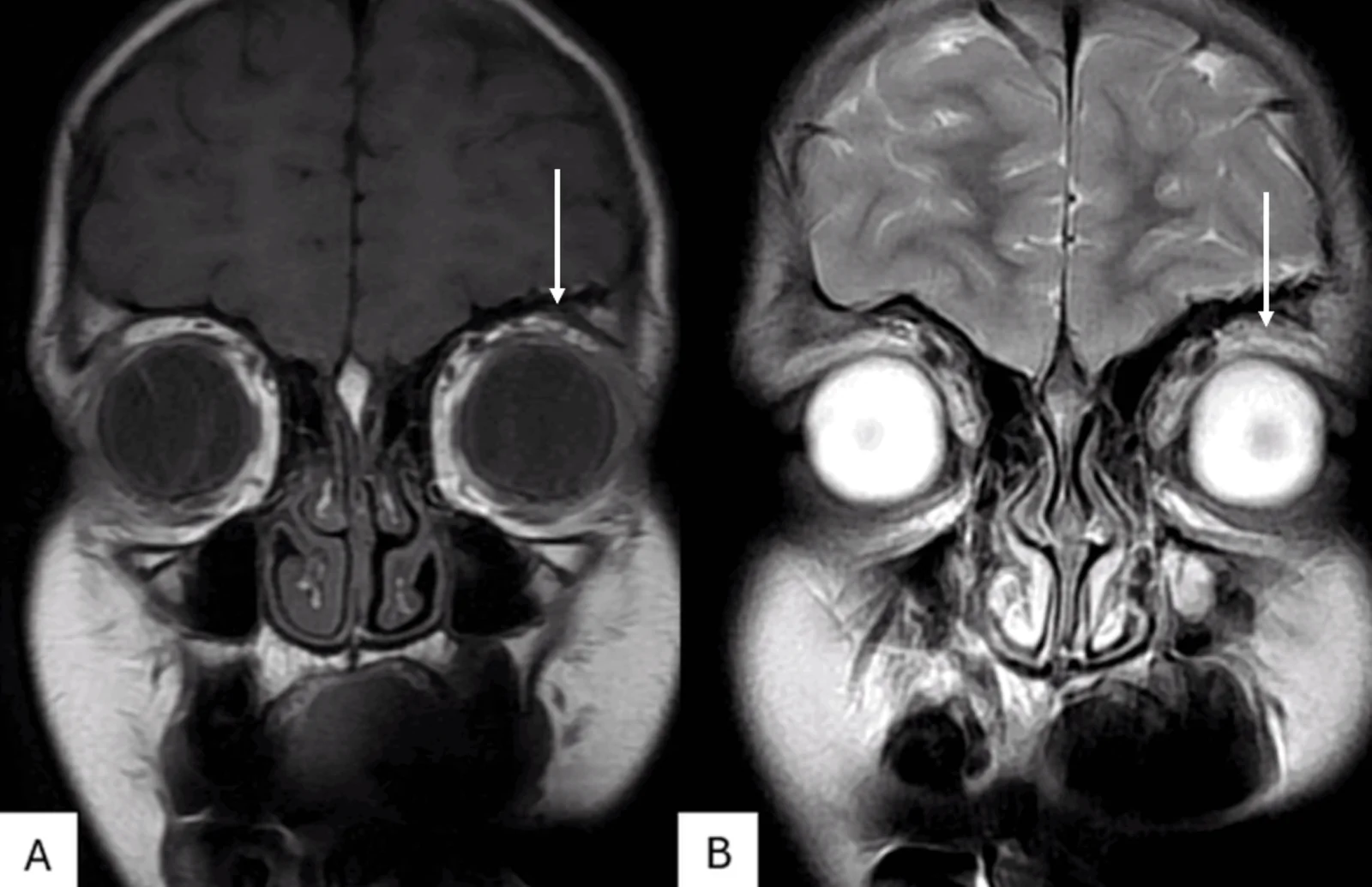
Postoperative magnetic resonance imaging of the head and orbits 16 months after surgical intervention. (A) Coronal T1-weighted image and (B) coronal T2-weighted image demonstrating stable changes of left superior orbitotomy with associated artifact, no evidence of residual osseous lesion, and no evidence of new lesions.

## Discussion

The present case illustrates the diagnostic difficulties associated with pediatric orbital LCH. The patient was initially evaluated for suspected orbital cellulitis due to characteristic eyelid swelling and erythema. Subsequent MRI findings, along with normal inflammatory markers and absence of fever, shifted suspicion toward a non-infectious process, and the diagnosis was reached only after resection and histopathological examination. Langerin (CD207) immunostaining was not performed, although the diagnosis was supported by characteristic histopathology and CD1a and S100 positivity. The Ki-67 proliferation index was 40%, consistent with high proliferative activity; however, Ki-67 has not been established as an independent prognostic marker in LCH [16]. Diagnosis of orbital LCH remains challenging due to its highly variable presentation, symptom overlap with more common infectious conditions, and overall low incidence [4,17,18]. Nonetheless, familiarity with common imaging findings and recognition of a poor or transient response to antibiotic therapy may enable faster diagnosis and improve patient outcomes, particularly when the lesion involves a CNS-risk location [4].

This case demonstrates several unusual features that expand upon the existing literature. As proptosis is typically expected secondary to mass effect of these lesions on the globe, an atypical finding was the presence of enophthalmos with hypoglobus in the affected eye. This displacement may reflect the position and direction of mass effect from the superolateral lesion. Furthermore, in our patient, intraoperative inspection demonstrated dural adherence. The adjacent meningeal enhancement on MRI was consistent with this operative finding but could also reflect reactive inflammation. However, neither enhancement nor adherence established histologically confirmed dural invasion. Previous reports of intracranial extension often rely on imaging findings such as dural tail signs on MRI without operative confirmation [4]. Only isolated cases and two patients in a single case series have been confirmed to have direct dural attachment [10-12]. Given that intracranial extension and dural involvement may influence CNS-risk classification and treatment decisions in orbital LCH, our case underscores the importance of carefully assessing and documenting the relationship of the lesion to the dura during surgery.

The case also highlights the importance of tailoring the intervention method for orbital LCH based on clinical course and the extent of disease. Orbital LCH is often managed with less invasive methods, such as curettage or intralesional steroids, and many bony defects are left to resolve spontaneously [5,6]. Although diagnostic biopsy followed by treatment planning is an accepted alternative for calvarial LCH lesions, the extent of orbital roof involvement, associated globe mass effect, suspected intracranial extension, and recurrent clinical swelling prompted the multidisciplinary team to proceed directly to resection in this case. Confirmatory biopsy followed by observation represents a reasonable alternative strategy, although it is best established for calvarial rather than orbital lesions [9,13,14]. Thus, the challenge of managing these cases extends beyond diagnosis to the choice between conservative and operative management. Additionally, resection resulted in a full-thickness orbital roof defect greater than 1 cm with loss of the normal anatomic boundary between the orbit and intracranial compartment. Given the absence of a definitive intraoperative diagnosis and concern for maintaining structural separation and preventing herniation of intracranial contents into the orbit, the multidisciplinary team elected to perform rigid reconstruction.

To our knowledge, titanium mesh reconstruction of the orbital roof following orbital LCH resection in a pediatric patient has not previously been reported. A targeted PubMed search using combinations of LCH-related terms and orbital reconstruction terminology identified no prior report specifically describing this reconstructive approach. Although titanium mesh provides rigid reconstruction and avoids donor-site morbidity, its use in pediatric patients raises unique long-term considerations because the implant does not accommodate continued craniofacial growth and may be associated with hardware migration [19]. Retained titanium hardware may also become palpable or painful, requiring subsequent removal [19]. When utilized for orbital roof repair specifically, long-term orbital asymmetry and implant displacement have been reported following titanium mesh reconstruction in a young child, underscoring the potential impact on continued craniofacial development [20]. However, larger pediatric series of titanium mesh cranioplasty have demonstrated generally favorable long-term outcomes, with low rates of implant-related complications and no clear evidence of growth restriction [21,22]. Accordingly, continued clinical and radiographic follow-up is warranted to assess craniofacial growth, implant position, and the potential need for future surgical revision. Separate long-term craniofacial surveillance related to the titanium reconstruction is planned at two-year intervals to assess implant position and craniofacial growth, and routine implant removal is not planned in the absence of implant-related complications. Our experience illustrates that more extensive surgical management may be considered in selected pediatric patients with orbital LCH when substantial structural erosion, mass effect, and suspected dural adherence or involvement are present.

A postoperative systemic chemotherapeutic regimen of vinblastine and prednisone was initiated because the lesion demonstrated intracranial extension with operative dural adherence, features associated with CNS-risk orbital LCH [6,23,24]. CNS-risk lesions have been associated with an increased risk of diabetes insipidus (DI) and subsequent neurodegenerative complications of LCH [4]. However, controversy remains regarding whether systemic therapy is required for the initial presentation of unifocal bone LCH, even when CNS-risk bones are involved [25]. There is evidence that unifocal lesions with orbital and intracranial extension may resolve without systemic chemotherapy; however, these findings have yet to be validated in larger cohorts [26]. In the present case, systemic chemotherapy was selected following multidisciplinary evaluation, and the patient remained without recurrence or DI during the reported follow-up period.

As a single case report, this study cannot establish the comparative benefits of surgical resection, rigid reconstruction, or systemic therapy relative to less invasive approaches and should not be interpreted as establishing a new standard of care. Additional limitations include the relatively limited duration of follow-up and incomplete molecular characterization beyond negative BRAF V600E testing.

## Conclusions

This case represents one of the few reports with operative confirmation of dural adherence and illustrates one potential management strategy for pediatric orbital LCH with substantial mass effect, orbital roof destruction, and intracranial extension. In this selected patient, surgical resection with orbital roof reconstruction and systemic chemotherapy was associated with a favorable outcome. However, management strategies for orbital LCH remain variable, and successful conservative approaches have also been reported. As a single case report, these findings cannot establish indications for aggressive intervention. Additional cases with long-term follow-up are needed to better define the roles of surgery, reconstruction, and systemic chemotherapy in patients with intracranial or dural involvement.
